# Temporal changes in sphingolipids and systemic insulin sensitivity during the transition from gestation to lactation

**DOI:** 10.1371/journal.pone.0176787

**Published:** 2017-05-09

**Authors:** J. Eduardo Rico, Sina Saed Samii, Alice T. Mathews, Jacqueline Lovett, Norman J. Haughey, Joseph W. McFadden

**Affiliations:** 1 Division of Animal and Nutritional Sciences, West Virginia University, Morgantown, West Virginia, United States of America; 2 Department of Neurology, Johns Hopkins University School of Medicine, Baltimore, Maryland, United States of America; Universite Paris Diderot-Paris7 - Batiment des Grands Moulins, FRANCE

## Abstract

Reduced insulin action develops naturally during the peripartum to ensure maternal nutrient delivery to the fetus and neonate. However, increased insulin resistance can facilitate excessive lipolysis which in turn promotes metabolic disease in overweight dairy cattle. Increased fatty acid availability favors the accumulation of the sphingolipid ceramide and is implicated in the pathogenesis of insulin resistance, however, the relationship between sphingolipid metabolism and insulin resistance during the peripartum remains largely unknown. Our objectives were to characterize temporal responses in plasma and tissue sphingolipids in lean and overweight peripartal cows and to establish the relationships between sphingolipid supply and lipolysis, hepatic lipid deposition, and systemic insulin action. Twenty-one multiparous lean and overweight Holstein cows were enrolled in a longitudinal study spanning the transition from gestation to lactation (d -21 to 21, relative to parturition). Plasma, liver, and skeletal muscle samples were obtained, and sphingolipids were profiled using LC/MS/MS. Insulin sensitivity was assessed utilizing intravenous insulin and glucose challenges. Our results demonstrated the following: first, insulin resistance develops postpartum concurrently with increased lipolysis and hepatic lipid accumulation; second, ceramides and glycosylated ceramides accumulate during the transition from gestation to lactation and are further elevated in overweight cows; third, ceramide accrual is associated with lipolysis and liver lipid accumulation, and C16:0- and C24:0-ceramide are inversely associated with systemic insulin sensitivity postpartum; fourth, plasma sphingomyelin, a potential source of ceramides reaches a nadir at parturition and is closely associated with feed intake; fifth, select sphingomyelins are lower in the plasma of overweight cows during the peripartal period. Our results demonstrate that dynamic changes occur in peripartal sphingolipids that are influenced by adiposity, and are associated with the onset of peripartal insulin resistance. These observations are in agreement with a putative potential role for sphingolipids in facilitating the physiological adaptations of peripartum.

## Introduction

Major physiological adaptations develop as dairy cattle transition from late gestation to early lactation as a means to ensure an uninterrupted supply of nutrients to the prenatal fetus and newborn offspring. During the last trimester of gestation, the growth of the conceptus follows an exponential curve increasing demand for glucose by the gravid uterus [1, 2]. Immediately following parturition, the synthesis of milk and its components can double daily energy requirements in high-producing dairy cows [3]. Moreover, increased glucose flux toward the mammary gland favors lactose synthesis and results in a three to four-fold increase in daily glucose requirements [4]. Two important metabolic adaptations that facilitate glucose partitioning toward the fetus and the mammary gland include a progressive reduction of insulin secretion by the pancreas, and impaired insulin responsiveness in adipose and skeletal muscle tissues [4, 5]. The progressive development of insulin resistance during peripartum has been validated through direct measurements of insulin secretion and responsiveness, such as glucose tolerance testing and insulin tolerance testing [6, 7, 8]. Because insulin is an anti-lipolytic hormone, the excessive mobilization of fatty acids from adipose tissue is an unfavorable outcome of reduced insulin action. In turn, fatty acids function as substrate for hepatic triacylglycerol synthesis and ketogenesis which predispose peripartal dairy cows to metabolic disease (i.e. fatty liver and ketosis), compromised milk production, and infertility.

A key mechanism that regulates the progression of insulin resistance in monogastrics involves the dysregulation of sphingolipid metabolism and the accrual of bioactive ceramide [9]. In addition to their roles as signaling molecules regulating cell growth, apoptosis, and vesicular trafficking [10], ceramides can also contribute to systemic insulin resistance by disrupting intracellular insulin signaling [11, 12]. The definitive mechanism of insulin antagonism is dependent on the involved tissue, sphingolipid pathway, cellular compartment, ceramide moiety, as well as inflammatory and nutritional status [13, 14, 15]. For instance, excess palmitic acid increases the de novo synthesis and lipoprotein export of ceramides in the liver [16, 17], contributing to systemic and skeletal muscle-specific insulin resistance, and inflammation [18]. The induction of sphingomyelinase activity by pro-inflammatory tumor necrosis factor-α can also contribute to ceramide synthesis [19] and insulin resistance [20]; however, the ability of sphingomyelin hydrolysis to influence the circulating ceramide pool is uncertain. Alternative to the role of extracellular ceramide, the intracellular synthesis of ceramide antagonizes insulin sensitivity in adipocytes [14]; however, the role of ceramide as a mediator of skeletal muscle insulin resistance has been contested [21, 22]. Although the ceramide-mediated mechanisms of insulin resistance are being defined, circulating ceramides have emerged as prognostic biomarkers for the development of insulin resistance, dyslipidemia, and nonalcoholic fatty liver disease in overweight subjects [23, 24]. Importantly, to date, the metabolic roles of ceramides during the maternal transition from gestation to lactation and their relation to systemic insulin sensitivity remain largely unknown.

We recently demonstrated that estimated insulin sensitivity is decreased in peripartal dairy cows experiencing a progressive accumulation of fatty acids and ceramide in plasma, a response augmented by increased adiposity prepartum [25]. With the understanding that insulin sensitivity gradually improves in dairy cattle as lactation and fetal development advances [5], we established that circulating ceramides concomitantly decline in pregnant dairy cows during mid-lactation, a response that can be diminished by feeding palmitic acid [26]. Our research group has proposed that ceramide may be an intrinsic mediator of adaptive metabolism that accompanies the onset of lactation and facilitates increased glucose partitioning for the mammary synthesis of milk [26]. However, our understanding of peripartal sphingolipid metabolism in ruminants is currently limited to changes in circulating ceramides, and no characterization of hepatic supply and peripheral accumulation of ceramides are available to date. Moreover, considering that surrogate indices to estimate insulin sensitivity in ruminants should be interpreted with caution, there is a necessity for direct measures of systemic insulin sensitivity that can be used to evaluate its association with sphingolipids during the peripartum period. Therefore, the aim of this study was to expand our understanding of bovine sphingolipid metabolism, and glucose and insulin tolerance during the periparturient period. Specifically, we wanted to assess changes in ceramide, monohexosylceramide (GlcCer), lactosylceramide (LacCer), and sphingomyelin concentrations in cows with a lean or overweight phenotype during late pregnancy. Our second aim was to compare alterations in sphingolipid compartments with the extent of hepatic lipid deposition and systemic insulin action. Our attention was focused on C16:0- and C24:0-ceramide, the two major moieties found in bovine plasma [25, 26], because of their associative and causative roles in the development of insulin resistance in monogastrics [15, 18, 27].

## Methods

### Experimental design

Experimental procedures were approved by the West Virginia University Institutional Animal Care and Use Committee (Morgantown, WV). The experiment was completed at Dovan Farms, a West Virginia University Agricultural Research and Education Partner and a commercial dairy farm (Berlin, PA). Nonlactating, pregnant, multiparous Holstein cows (n = 21) were enrolled 28 d before expected parturition and allocated according to one of two groups based on their adiposity as either lean or overweight with a body condition score (BCS; 1.0 to 5.0 scale [28]) of 2.93 ± 0.16 and 4.01 ± 0.20 BCS, respectively. Cows were housed in a straw-bedded pen and trained to access feed by using Calan gate feeders (American Calan, Inc., Norwood, NH). Because of management constraints of the commercial operation, cows were moved to adjacent free-stalls starting on d 7 of lactation. Body weights and BCS were recorded weekly. Body condition score was independently recorded by three trained investigators and data were averaged for each cow pre and postpartum. Diets were formulated to meet nutrient recommendations [29], and were provided once and twice daily during gestation (0900 h) and lactation (0900 and 1600 h), respectively. Cows were fed at 110% of expected intake which was adjusted daily when monitored. Cows were provided free access to water at all times. Access to feed was blocked from 0700 to 0900 h to allow for collection of orts and weighing of feed. Samples of total mixed rations were obtained weekly throughout the experiment. Cows were milked twice daily (0600 and 1700 h) through d 6 of lactation, then thrice daily (0430, 1230 and 2030) thereafter. Milk samples were collected on d 1 through 7, and d 10, 14, 21 and 28 of lactation.

### Blood and tissue collection

Basal blood collection (10 mL) by coccygeal venipuncture occurred at 0600 h on d -28, -21, -7, and -5, relative to expected parturition, and d 0, 2, 4, 7, 14, and 21 postpartum. Blood was kept on ice for 30 min until centrifugation at 3,400 × g for 10 min. Following centrifugation, plasma was removed and snap-frozen in liquid nitrogen and stored at -80°C until further analysis.

Liver and skeletal muscle biopsies were performed prior to morning feeding on d -21 and -7, relative to expected parturition, and d 4 of lactation using aseptic techniques. Cows were anesthetized using xylazine (0.04 mL/kg of body weight) delivered via the coccygeal vein. After the hair was clipped, biopsy sites were sanitized with iodine scrub and anesthetized with lidocaine HCl (5 mL; Vedco Inc., Saint Joseph, MO) delivered subcutaneously. For liver biopsies, the 11^th^ intercostal space was prepared by making a 0.5 cm incision through the skin and a fabricated trocar was utilized to collect approximately 1 g of liver tissue [30]. Skeletal muscle biopsies were performed by making a 0.5 cm incision in the skin adjacent to the semitendinosus muscle. Approximately 500 mg of muscle tissue was collected using a Bard Magnum biopsy instrument mounted with a 12 gauge needle (Bard Biopsy Systems, Tempe, AZ). Tissue was snap-frozen in liquid nitrogen and stored at -80°C. Following the collection of tissue, biopsy sites were stapled and sprayed with antiseptic. Immediately following each biopsy, antibiotic was delivered once by intramuscular injection (0.68 mL/kg of body weight; Excenel RTU; Zoetis, Florham Park, NJ) in accordance with the Institutional Animal Care and Use Committee.

### Insulin and glucose challenges

A jugular catheter was inserted 24 h prior to insulin and glucose challenges, and patency was maintained by flushing with heparinized saline every 10 h. Access to feed was blocked 2 h prior to testing. Insulin challenges were performed at 1500 h on d -26 and -13 relative to parturition, and d 5 of lactation using previously described methods [31]. Cows were intravenously infused with insulin (0.1 IU of insulin/kg of body weight; Humulin R; Eli Lilly Co., Indianapolis, IN) that was followed by a saline solution flush (10 mL). Glucose challenges were performed at 1500 h on d -25 and -12 relative to parturition, and d 6 of lactation, using previously established methods [31]. Cows were intravenously infused with 300 mg of glucose (dextrose, 50% wt/vol) per kg of BW, followed by a saline solution flush (10 mL). For both tests, blood samples (10 mL) were collected at -10, 0, 10, 20, 30, 40, 60, 90, 120, 150, and 180 min relative to insulin or glucose infusion. Blood was processed as described above. Access to feed was allowed immediately following sample collection.

### Sample analyses

Dry matter content of the mixed ration was determined daily by drying at 60°C until a static weight was observed. Mixed rations were composited twice monthly and analyzed for NDF with heat-stable α-amylase and sodium sulfite [32], CP (method 990.03) [33], and starch [34] (Table 1; Cumberland Valley Analytical Services, Inc.; Cumberland, MD). Individual milk samples were analyzed for fat, true protein, lactose, and milk urea nitrogen concentrations using mid-infrared spectroscopy within 1 wk of collection (method 972.160; Dairy One, Hagerstown, MD) [35].

**Table 1:** Ingredients and nutrient composition of diets fed to lean and overweight cows during the transition from late pregnancy to early lactation.

Ingredients, % of DM	Diets	
	Prepartum	Postpartum
Corn silage	30.7	42.4
Mixed grass haylage	28.4	7.0
Mixed grass hay	13.2	2.1
Dry ground corn	–	14.4
Prepartum mix^1^	13.9	–
Lactation mix A^2^	–	14.0
Soybean meal	9.0	4.7
Cottonseed with lint	–	4.5
Sugar cane syrup	–	3.6
Lactation mix B^3^	–	4.5
Close-up supplement^4^	4.8	2.7
Rumensin^5^	–	0.1
**Nutrient, % of DM**		
Dry matter (% as-fed)	55.5	51.6
Neutral detergent fiber	48.3	37.6
Acid detergent fiber	32	25.2
Crude protein	12.2	16.5
Starch	14.1	21.2
Ether extract	3.2	4.3
Ash	7.5	7.3

^1^Mix contained 27% commercial dry cow mix with Animate (Phibro Animal Health Corp., Teaneck, NJ), 17.3% ground corn, 17.3% crimped oats, 13% corn distillers, 12.9% soybean meal, 4.3% calcium carbonate, 4.3% calcium sulfate, 2.6% Omnigen AF (Phibro Animal Health Corp.), 1.1 Monocalcium phosphate, and < 1% of each of the following: Sel-plex 600 (Alltech Biotechnology, Nicholasville, KY) and vitamin E.

^2^Mix contained 33.6% citrus pulp, 19% soybean meal. 15.6% canola meal, 14% soybean hulls, 4.5% calcium carbonate, 4.5% sodium bicarbonate, 2.8% urea, 2.7% fat, 2.4% sodium chloride, and <1% from each of the following: Monocalcium phosphate, biotin, and Rumensin 90 (Elanco Animal Health, Greenfield, IN).

^3^Mix contained 43.6% Fermenten (Church and Dwight Co., Princeton, NJ), 21.8% calcium carbonate, 10.8% soybean hulls, 7.8% Mintrex blend (Novus International Inc., St. Charles, MO), 5.2% blood meal, 3.5% magnesium oxide, 2.6% Celmanax (Vi-COR, Mason City, IA), 2.6% Omnigen (Phibro Animal Health Corp.), and <1% of each of the following: vitamin E, selenium selenite, and selenium yeast 600.

^4^Mix contained 74.5% ground oats, 15.3% commercial amino acid, 8% Reashure (Balchem Encapsulates, Slate Hill, NY), and <1% of each of the following: vitamin E and Niashure (Balchem Corporation, New Hampton, NY).

^5^Rumensin for dairy included at 4,890 mg/kg.

Plasma concentrations of glucose, fatty acids, and β-hydroxybutyrate (BHB) were determined by enzymatic methods using commercial kits (Autokit Glucose, HR Series NEFA-HR (2) and Autokit 3-HB, respectively; Wako Chemicals USA, Inc., Richmond, VA). Plasma concentrations of insulin were determined by immunoassay (Mercodia Bovine Insulin ELISA; Mercodia AB, Uppsala, Sweden). All spectrophotometric measurements were conducted using a SpectraMax Plus 384 Microplate Reader (Molecular Devices, Sunnyvale, CA). All intra- and interassay coefficient of variations were <5 and <10%, respectively, for plasma glucose, fatty acids, BHB, and insulin. Hepatic liver lipid was extracted and quantified using the methodology described by Starke and collaborators [36].

Ceramide extraction was conducted using previously established methods that employ a modified Bligh and Dyer procedure [26, 37]. Prior to analysis, the organic layers from plasma and tissue extracts were dried under nitrogen (Organomation Associates Inc., Berlin, MA) and re-suspended in pure methanol. Ceramides were detected by multiple reaction monitoring using a liquid chromatography, electrospray ionization, tandem mass spectrometry system (API3000; AB Sciex Inc., Thornhill, ON, Canada) in positive mode. Liquid chromatography and MS/MS parameters have been previously described [25]. Differences in extraction efficiency and fluctuations in mass spectrometer efficiency were normalized to the C12:0-ceramide internal standard. Instrument efficiency was monitored daily and individual plasma extracts were re-analyzed if the internal standard deviated more than 30% from the overall median internal standard value. The original or re-run data with internal standard concentrations closest to the median C12:0-ceramide value were included in the final analysis. Plasma ceramide concentrations were determined by fitting the identified ceramide species to standard curves based on acyl-chain length. Ceramide standards were purchased from Sigma-Aldrich (C16:0, C18:0, and C18:1; St. Louis, MO) or Avanti Polar Lipids (C20:0, C22:0, C24:0, and C24:1; Alabaster, AL). Glycosylated ceramides were purchased from Matreya Inc. (C24:0-GlcCer, C16:0-LacCer, C18:0-LacCer; Pleasant Gap, PA). Instrument control and quantitation were performed using Analyst 1.4.2 and MultiQuant software (AB Sciex Inc.), respectively.

### Calculations and statistical analysis

Changes in BCS and body weight of lean and overweight cows were calculated as the differences between values at d -21 and 21, relative to parturition, and were analyzed using the GLM procedure of SAS (version 9.3; SAS Institute Inc., Cary, NC). All data for time-dependent changes in plasma, tissue and milk variables were analyzed as repeated measures over time relative to parturition under the MIXED procedure of SAS. The statistical model included the random effect of cow nested within BCS, the fixed effects of BCS, day relative to parturition, and their interaction. The covariance structure was modeled to identify patterns that best describe relationships between the repeated measures. The most appropriate covariance structure was selected for each response variable after evaluating 3 different covariance structures (variance components, first-order autoregressive, and compound symmetry), and the structure with the smallest Akaike’s information criterion coefficient was selected for analysis. The method of Kenward-Roger was used for calculation of denominator degrees of freedom. The SLICE option of SAS was used to compare treatment differences at individual time points.

Parametric Pearson correlations were performed to determine variables of interest. Relationships between select ceramides were evaluated by regression analysis using the REG procedure of SAS. The full model included the random effect of cow and the linear and quadratic effects of each predictor. Studentized residual values >3.0 or <-3.0 were considered outliers and removed from the analysis (typically 1 per response variable). All results are expressed as least squares means and their standard errors, unless stated otherwise. Significance was declared at *P* < 0.05 and tendencies at *P* < 0.10. For presentation of plasma sphingomyelin data, heat map was generated using MetaboAnalyst 3.0 [38].

## Results and discussion

Although twenty-one prepartum cows were enrolled, seven cows were not included in our analyses because they were diagnosed with a metabolic disease and subsequently treated by the herd veterinarian; therefore, our data represents fourteen cows that transitioned from gestation to lactation without clinical interventions. Animal performance and metabolic responses for lean and overweight cows are presented in Table 2. At enrollment, body weight was comparable; however, overweight cows displayed an increased BCS, relative to lean cows. Elevated adiposity prepartum predisposed overweight cows to greater BCS and body weight loss during the peripartum. The dramatic loss of deposited fat in overweight cows was accompanied by suppressed appetite and elevated milk fat yield postpartum, responses that have been previously observed in dairy cattle with overnutrition syndrome [39, 40].

**Table 2:** Animal performance and metabolic responses during peripartum.

	Prepartum			Postpartum			ANOVA *P*-value^1^		
	Lean	Overweight	SEM	Lean	Overweight	SEM	BCS	Stage	BCS × Stage^2^
**Animal performance**									
BCS, units	2.91	4.00***	0.07	2.59	3.30***	0.08	<0.001	<0.001	<0.01
Body weight, kg	642	679	18	658	691	18.2	0.12	<0.01	0.88
BCS change, units^3^	-	-	-	-0.39	-0.91	0.10	<0.01	-	-
Body weight change, kg^3^	-	-	-	-63.2	-141	12.4	<0.001	-	-
Dry matter intake, kg/d	11.2	10.3	1.43	16.0	11.0*	1.53	<0.001	<0.001	<0.01
Milk yield, kg/d^4^	-	-	-	27.80	26.4	3.57	0.78	-	-
Fat yield, kg/d	-	-	-	0.83	1.59	0.15	<0.01	-	-
Protein yield, kg/d	-	-	-	0.89	0.91	0.14	0.95	-	-
Lactose yield, kg/d	-	-	-	1.13	1.10	0.19	0.89	-	-
**Metabolic responses**									
Glucose, mg/dL	68.0	65.8	3.29	58.7	59.6	3.29	0.87	<0.01	0.50
Insulin, μU/mL	7.13	14.8*	2.55	5.17	7.50	2.45	0.15	<0.01	0.07
Fatty acids, mmol/L	0.24	0.42†	0.07	0.49	1.02***	0.07	<0.001	<0.001	0.02
Liver lipid, % wt/wt	4.02	6.71*	0.80	5.80	13.9***	0.93	<0.001	<0.001	<0.01
BHB, μmol/L^5^	318	345	21.6	344	419	19.8	0.04	0.02	0.23
GSRN, % of basal^6^	-71.0	-60.0	0.05	-67.0	-57.0	0.05	<0.05	<0.05	0.55
ISRG, mg/dL^7^	-30.8	-34.3	3.9	-25.8	-26.2	3.7	0.27	<0.001	0.64

^1^Significance of main effects modeled. Body condition score (BCS) indicates adiposity score as either lean or overweight, and stage indicates pre or postpartum.

^2^Difference between lean and overweight cows when significance or tendency for a group × stage interaction was detected; ***, *P* < 0.001; **, *P* < 0.01; *, *P* < 0.05; †, *P* < 0.10.

^3^Change in BCS or body weight as calculated as the difference between d 21 and d -21, relative to parturition.

^4^Milk yield and components are average of first 3 weeks postpartum when recorded and measured.

^5^BHB = β-hydroxybutyrate.

^6^GSRN = glucose-stimulated reduction of plasma fatty acids following a glucose challenge calculated as minimum minus basal plasma nonesterified fatty acids.

^7^ISRG = insulin-stimulated reduction of glucose after 40 min of insulin challenge start calculated as change from basal.

Maternal adaptations of metabolism during peripartum ensure nutrient delivery to the developing fetus and neonate [41]. Indeed, peripartal reductions in insulin secretion and the development of peripheral insulin resistance constitute key adaptations of metabolism that facilitate increased glucose delivery to the fetus and the mammary gland, which result in increased adipose tissue lipolysis [4, 5, 41]. However, accelerated lipolysis can be a detriment to cow health and performance [42, 43]. As anticipated, circulating fatty acids increased in all cows postpartum with a concomitant decrease in plasma insulin and glucose levels. Cows with greater adiposity prepartum are at enhanced risk for developing a postpartum metabolic disease [39, 40]. Comparable to our previous work [25], circulating insulin and fatty acids were elevated in overweight cows. Because of the anti-lipogenic role of insulin, lipolysis-derived fatty acids are expected to be low in healthy individuals when insulin is elevated. The elevated fatty acid concentration in plasma of overweight cows during prepartum suggests these animal were experiencing adipose tissue insulin resistance, where above-normal insulin concentrations were insufficient to prevent lipolysis in overweight individuals.

The deposition of fat in liver is associated with the development of insulin resistance [44, 45], and precedes the development of metabolic disease in dairy cattle [46]. An increase in fatty acid supply was accompanied by a linear increase in liver lipid content during the peripartum (*r* = 0.91; *P* < 0.001). Accordingly, plasma BHB concentrations were elevated in overweight cows during the peripartum. The greater reduction in dry matter intake recorded in overweight cows certainly contributed to the severity of the observed metabolic stress. However, insulin resistance promotes adipose tissue lipolysis in dairy cattle and a greater magnitude of insulin resistance in overweight animals may augment adipose tissue lipolysis, and trigger metabolic impairment. In the current study, insulin-stimulated reductions in circulating glucose were lower postpartum; however, systemic insulin sensitivity was not influenced by adiposity. Reflecting a homeorhetic adaptation to lactation, we did measure increased plasma fatty acids following a glucose challenge in postpartum cows. Moreover, glucose-stimulated reductions in fatty acids were suppressed in overweight cows, relative to lean cows. These results support localized decreases in adipose tissue insulin sensitivity in postpartum cows that display greater adiposity prior to parturition.

**Fig 1:**
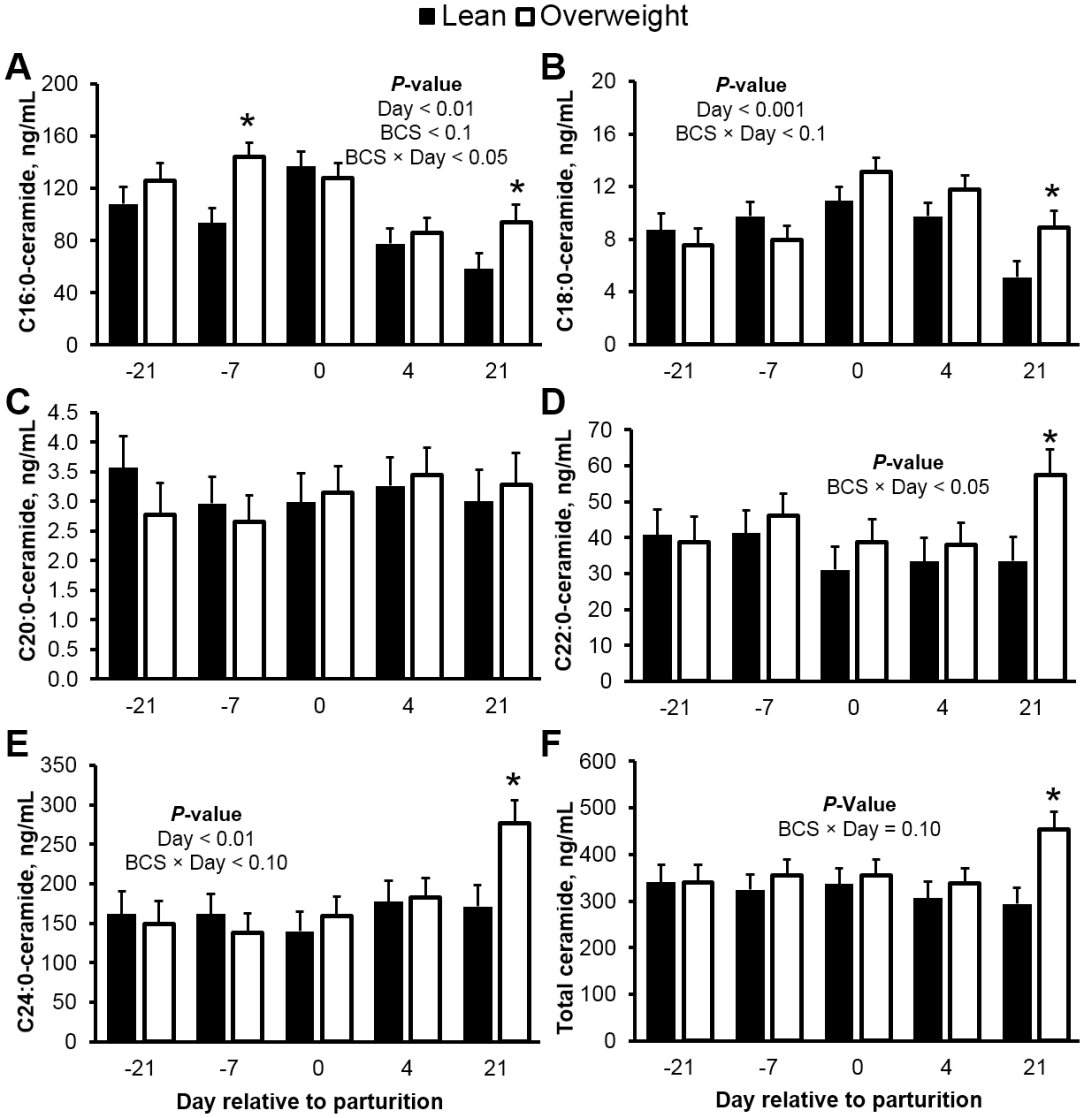
Plasma ceramides are elevated in overweight cows during the transition from gestation to lactation. Peripartal plasma concentrations of (**A**) C16:0-, (**B**) C18:0-, (**C**) C20:0-, (**D**) C22:0-, (**E**) C24:0-ceramide, and (**F**) total ceramide in lean and overweight dairy cows. Data are represented as least squares means and their standard errors. *, *P* < 0.05; †, *P* < 0.10. Total ceramide was calculated by summation of C16:0-, C18:0-, C20:0-, C22:0-, C22:1-, C24:0-, and C26:0-ceramide.

Ceramide is a putative mediator of insulin resistance in overweight monogastrics with type 2 diabetes or nonalcoholic fatty liver disease [23, 27, 47]. In our study, concentrations of circulating ceramide changed dynamically as cows transitioned from gestation to lactation (Fig 1). Representing an initial validation of our previous observations [25], total ceramide and predominant C24:0-ceramide in plasma were elevated in overweight cows postpartum (44 and 106%, respectively; *P* < 0.05), and these changes were related to fatty acid mobilization. For instance, plasma C24:0-ceramide was positively correlated with circulating fatty acid levels (*r* = 0.76; *P* < 0.01). In contrast, we observed a 38% decrease in C16:0-ceramide concentration at d 21, relative to d -21 prepartum (*P* < 0.01). However, similar to the C24:0 moiety, plasma C16:0-ceramide was higher in overweight cows at d -7 prepartum and d 21 postpartum. We and others have previously observed a reciprocal relationship between these abundant ceramides [25, 48], a response that is likely mediated by ceramide synthase 2 and 6, which control the acylation of sphinganine to produce C24:0- and C16:0-ceramide, respectively [48, 49]. Although not measured in the current study, we have recently shown that ceramide synthase 2 and 6 are expressed in the liver during lactation [26], in agreement with the predominance of their acylated products, C16:0- and C24:0-ceramide [49]. Recent evidence suggests that these two major ceramide moieties can independently promote insulin resistance [15, 18]. In support, circulating C24:0-ceramide was inversely associated with glucose clearance rates following an insulin challenge postpartum (*r* = -0.58; *P* < 0.05); however, this association was not observed for plasma C16:0-ceramide. Notably, we observed similar changes in circulating ceramides in mid-lactation dairy cattle fed a palmitic acid-enriched diet, which occurred with simultaneous elevations in milk yield [26, 50]. Similar to the adiposity-related changes in C16:0 and C24:0-ceramide, circulating C18:0- and C22:0-ceramide both increased in overweight cows by d 21 postpartum (Fig 1). Notably, in a recent study by Park and collaborators [51], C18:0-ceramide was the only ceramide to become elevated in skeletal muscle of obese mice with impaired systemic insulin tolerance. In our study, skeletal muscle C18:0-ceramide was inversely associated with insulin-stimulated reductions of glucose at d 4 postpartum (*r* = -0.67; *P* < 0.001). These observations support the notion that ceramides other than C16:0 and C24:0 could also be causal agents of insulin resistance. In contrast, we did not detect time or adiposity-associated changes in plasma C20:0-ceramide levels. This finding is in agreement with the observations from others [52] where no adiposity-related differences were found in circulating C20:0-ceramide concentrations and insulin sensitivity, supporting the observation that not all ceramide moieties are affected equally by the state of adiposity. In view that recent evidence [15, 18] demonstrates that 16:0- and C24:0-ceramide can disrupt insulin sensitivity (e.g. protein kinase B phosphorylation) in peripheral tissues, future work should investigate the role of circulating ceramides in the development of tissue-specific (e.g., adipose tissue and skeletal muscle) insulin resistance during peripartum.

**Fig 2:**
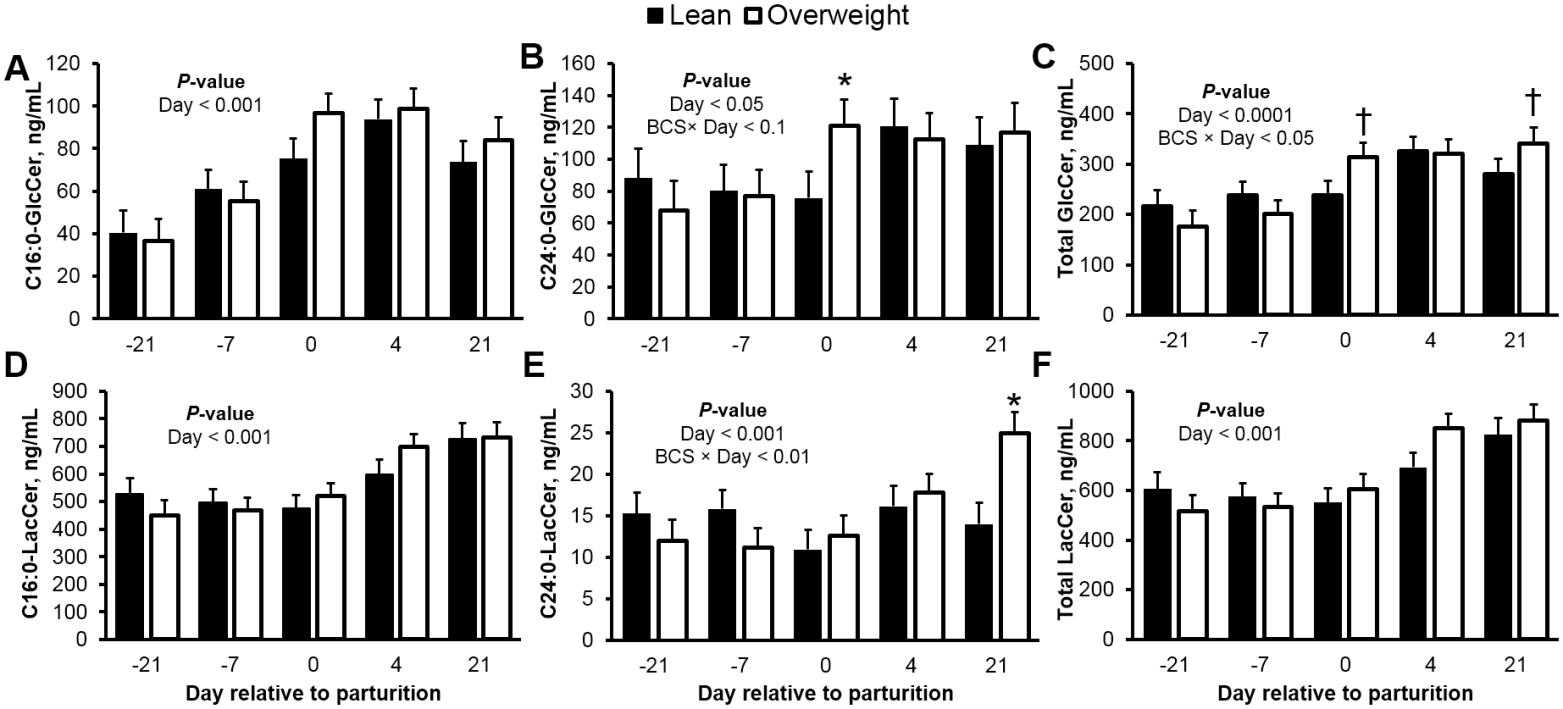
Glycosylated ceramides increase during peripartum and are elevated in overweight cows. Peripartal plasma concentrations (ng/mL) of monohexosylceramides (GlcCer), and lactosylceramides (LacCer) in lean and overweight cows. Plasma (**A**) C16:0-GlcCer, (**B**) C24:0-GlcCer, (**C**) total GlcCer, (**D**) C16:0-LacCer, (**E**) C24:0-LacCer, and (**F**) total LacCer. Data are represented as least squares means and their standard errors. *, *P* < 0.05; †, *P* < 0.10. Total GlcCer was calculated by summation of C16:0-, C16:1-, C18:0-, C18:1-, C20:0-, C22:0-, C22:1-, C24:0-, C24:1-, and C26:0-GlcCer. Total LacCer was calculated by summation of C16:0-, C18:0-, C22:0-, C24:0-, and C24:1-LacCer.

The insulin receptor can co-localize with complex glycosphingolipids in plasma membrane caveolae [53]. Specifically, gangliosides can disrupt insulin signaling by displacing the insulin receptor from these microdomains [53, 54]. Precursors for ganglioside synthesis include GlcCer and LacCer, and the pharmacological inhibition of GlcCer synthase can enhance insulin sensitivity and restore normoglycemia [55]. The concentrations of total GlcCer and LacCer in plasma increased as cows entered lactation (Fig 2), a response that developed with increased fatty acid supply (*r* = 0.49 and 0.50, respectively; *P* < 0.05) and adiposity. In a similar manner, we detected increased plasma concentrations of the predominant C16:0- and C24:0-GlcCer (83 and 54%, respectively; *P* < 0.05) over the course of the transition period, as well as increased concentrations of C16:1-, C20:0-, C22:1-GlcCer, and total GlcCer in overweight cows at d 21 postpartum (20, 46, 20, and 23%, respectively; *P* < 0.01). No effect of adiposity was detected for C16:0- and C22:0-GlcCer (S1 Fig). Interestingly, plasma concentrations of C18:0-GlcCer were consistently lower for overweight cows from d -21 to d 21 (*P* < 0.05; S1 Fig), however, the reason for this observation is unclear. Of note, an inverse relationship was observed between plasma C16:0-GlcCer and C16:0-ceramide (*r* = -0.40; *P* < 0.001), where the concentrations of C16:0-GlcCer increased progressively during the transition from gestation to lactation. From our repeated observations [25, 26], the active glycosylation of C16:0-ceramide by GlcCer synthase may contribute to its reduced concentration and to the reciprocal elevation of C16:0-GlcCer postpartum; however, changes in GlcCer synthase during the peripartum need to be characterized. Lactosylated ceramides in plasma increased by 53% from d -21 to 21 relative to parturition, a response driven by changes in C16:0-LacCer, which increased by 51%, and represented 85% of all quantified lactosylated ceramides. Similarly, C18:0-, C22:0-, C24:0-, and C24:1-LacCer increased during the peripartum period and were higher in overweight cows at d 21 postpartum (54, 42, 69, and 58%, respectively; S2 Fig). In support of an insulin antagonism role for glycosylated ceramides in adipose tissue [14], C16:0-GlcCer was positively associated with plasma fatty acids (i.e. area under the curve, and minimum and maximum fatty acid concentrations; *r* = 0.35 to 0.65; *P* < 0.01) and inversely related with glucose-stimulated reductions of fatty acids following a glucose challenge (glucose-stimulated reductions in fatty acids; *r* = -0.44; *P* < 0.01). In agreement with our observations, Park and collaborators [51] recently showed that C2C12 myocytes accumulate C16:0-GlcCer in response to the insulin resistance inducer, palmitic acid. However, studies evaluating the direct involvement of specific GlcCer moieties on the causation of insulin resistance remain limited.

**Fig 3:**
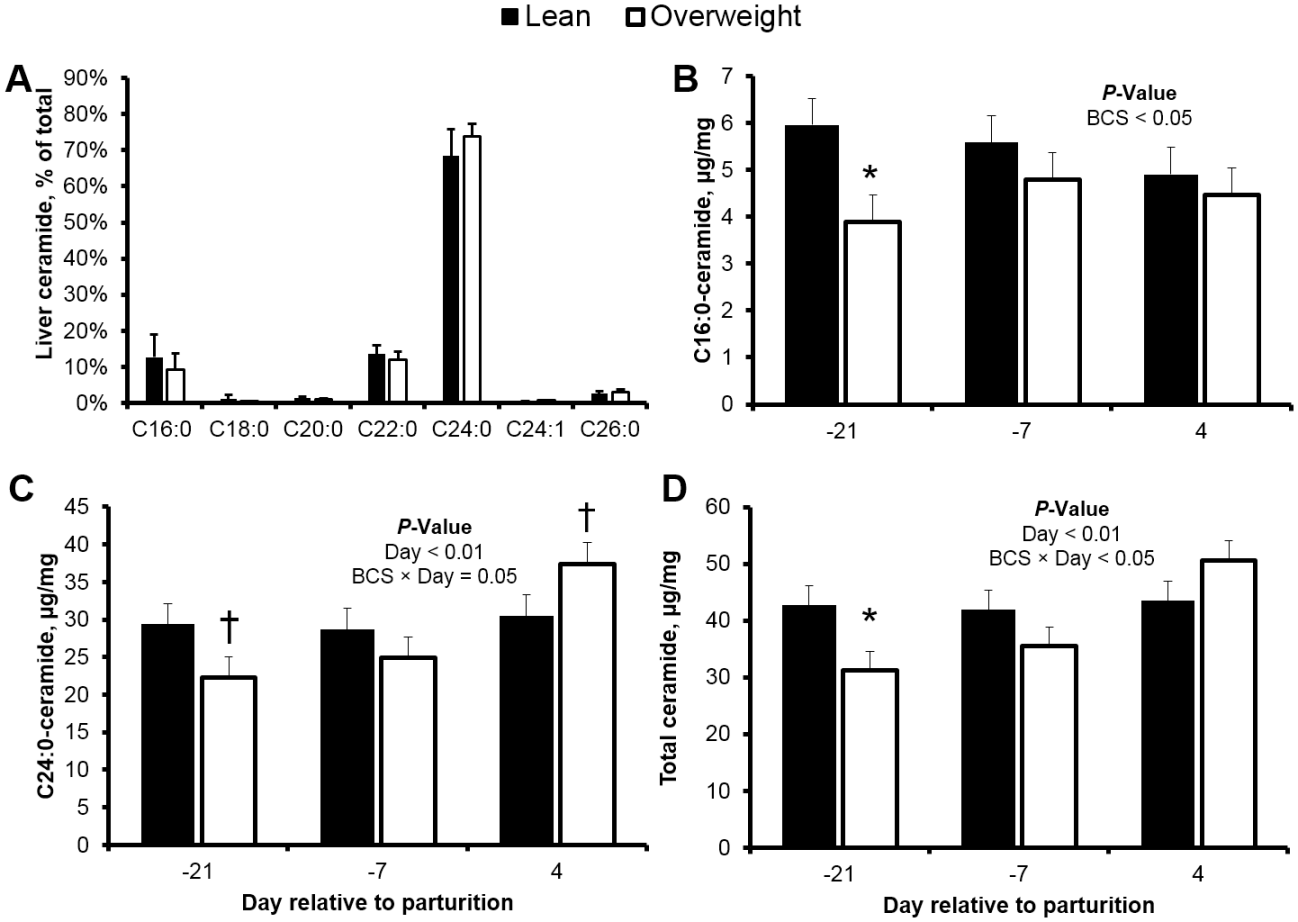
Total ceramide and C24:0-ceramide accumulate progressively in the liver of peripartal overweight cows. (**A**) Hepatic ceramide profile, and concentrations (μg/mg of wet liver) of (**B**) C16:0-, (**C**) C24:0-ceramide, and (**D**) total ceramide in peripartal lean and overweight cows. Data are represented as least squares means and their standard errors. *, *P* < 0.05; †, *P* < 0.10. Total ceramide was calculated by summation of C16:0-, C18:0-, C20:0-, C22:0-, C24:0-, C24:1-, and C26:0-ceramide.

**Fig 4:**
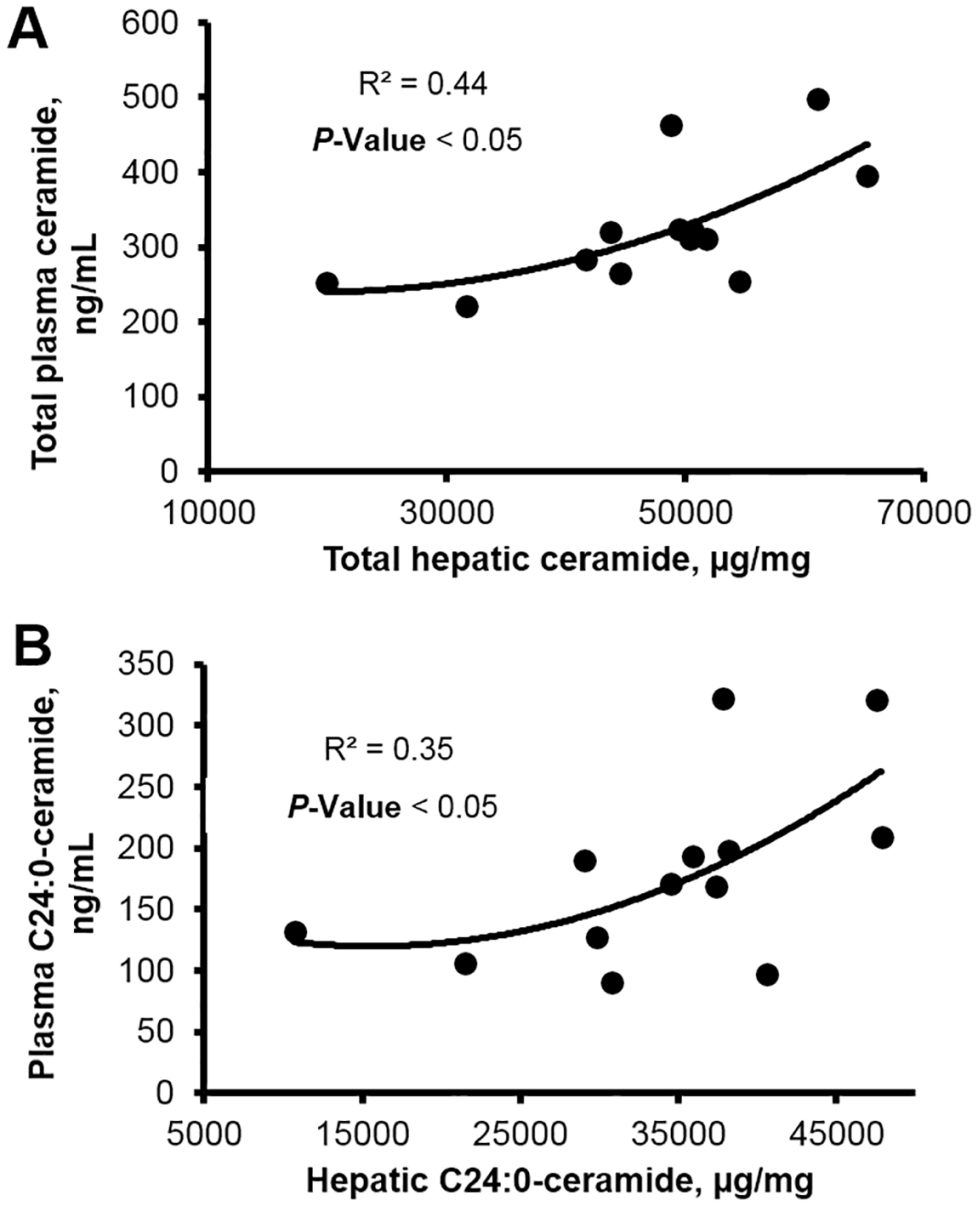
Plasma ceramide content is quadratically associated with hepatic ceramide accumulation postpartum. Regression analysis of plasma and liver concentrations of (**A**) total and (**B**) C24:0-ceramide in lean and overweight dairy cows at d 4 postpartum. No associations were detected prepartum. Total ceramide was calculated by summation of C16:0-, C18:0-, C20:0-, C22:0-, C24:0-, C24:1-, and C26:0-ceramide.

The hepatic de novo synthesis and lipoprotein export of ceramide during periods of increased fatty acid influx may contribute to insulin resistance [11, 14, 16]. Similar to our previous work that characterized the liver profile of ceramides in mid-lactation dairy cows [26], we observed that hepatic C24:0-ceramide was the predominant ceramide (50% of total ceramide), followed by C16:0- and C22:0-ceramide (Fig 3). Liver total ceramide and C24:0-ceramide progressively increased as overweight cows transitioned from gestation to lactation (~65%). Increased hepatic lipid accumulation in overweight cows was linearly associated with increased plasma fatty acids (*r* = 0.66; *P* < 0.01) indicating augmented fatty acyl-CoA supply for de novo ceramide synthesis. In support, regression analysis revealed a positive relationship between postpartum concentrations of hepatic and plasma C24:0-ceramide immediately postpartum (Fig 4); albeit no prepartum associations were found. Additionally, we observed an increase in C16:0-dihydroceramide in plasma (S3 Fig), an intermediate of the de novo synthesis pathway. Because we previously demonstrated that increased palmitic acid supply can increase hepatic C24:0-ceramide accumulation in dairy cattle [26], we conceive that palmitic acid derived from lipolysis may promote de novo ceramide synthesis during the peripartum. Interestingly, Xia and collaborators [56] showed that ceramide can contribute to hepatic steatosis by promoting protein kinase C-ζ activation and cluster of differentiation 36-mediated fatty acid uptake in liver. Moreover, ceramide can inhibit β-oxidation and increase triacylglycerol accumulation [48, 57] potentially by inducing cytochrome c release [58]. Therefore, hepatic ceramide accumulation during the peripartum may accelerate liver lipid accumulation in cows with augmented prepartum adiposity, and predispose these animals to insulin antagonism. In support, we detected positive associations between liver lipid content and hepatic ceramides (e.g. C24:0-ceramide, *r* = 0.40; *P* < 0.05).

In contrast to our observation of hepatic C24:0-ceramide, no time-associated changes were detected for hepatic C16:0-ceramide (Fig 3). Interestingly, C16:0-ceramide concentrations were lower in overweight cows at d -21, relative to lean, which was not simultaneously reflected in plasma. This constitutes an unexpected observation given that elevated concentrations of circulating fatty acids in overweight individuals would be expected to correspond to increased hepatic ceramide synthesis. The discrepancy may be explained by differences in the activity of the pathways contributing to ceramide breakdown and synthesis between lean and overweight cows prepartum (e.g., ceramidase or sphingomyelinase activity); however, no assertions in this regard can be made with our current data. Similarly, because our analysis of tissue ceramide accumulation and systemic insulin sensitivity was limited to the immediate postpartum (d 4), our ability to draw inferences on the origin of circulating ceramides and their effects on systemic insulin sensitivity are limited beyond this point (e.g. d 21 of parturition). Future studies should evaluate whether these relationships are sustained during the early postpartum and later in lactation, when lipolysis and ceramide synthesis from circulating fatty acids are expected to be minimal.

**Fig 5:**
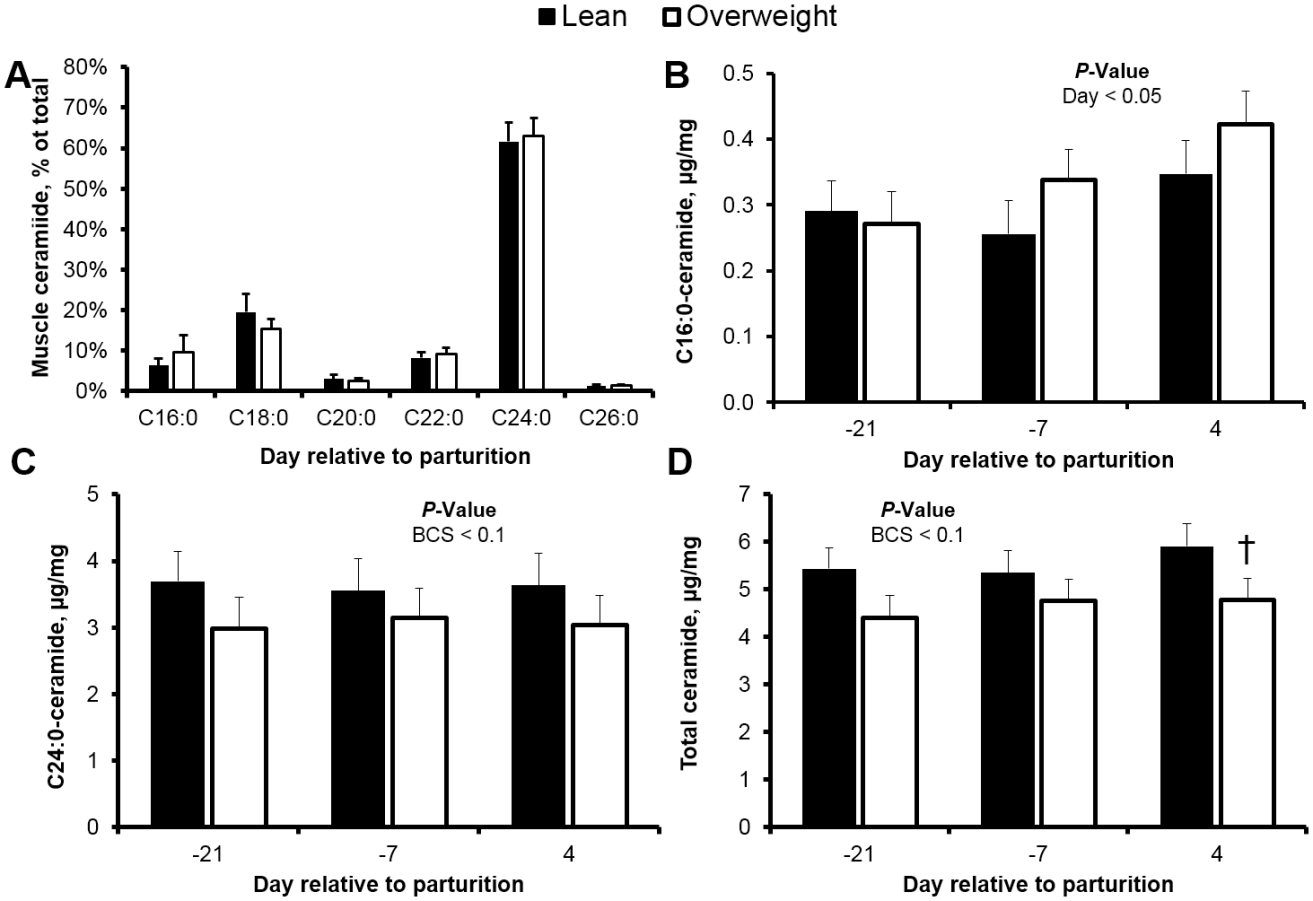
Skeletal muscle content of C16:0-ceramide increases progressively during peripartum. (**A**) Ceramide profile of skeletal muscle (μg/mg of wet skeletal muscle), concentrations of (**B**) C16:0-ceramide, (**C**) C24:0-ceramide, and (**D**) total ceramide. Data are represented as least squares means and their standard errors. †, *P* < 0.10. Total ceramide was calculated by summation of C16:0-, C18:0-, C20:0-, C22:0-, C24:0-, and C26:0-ceramide.

The complete mechanism by which ceramide inhibits insulin sensitivity in skeletal muscle tissue may depend on the origin of the sphingolipid. Different lines of evidence currently support the concept that circulating and intracellular ceramide can promote insulin resistance [18, 59, 60], and accumulate in tissues during type 2 diabetes [27, 61, 62]. However, the significance of ceramide accumulation during gestation and lactation has remained poorly defined. In our study, we characterized the ceramide content and profile of the peripartal bovine skeletal muscle, and identified C24:0-ceramide as the most abundant ceramide (Fig 5). The concentration of C24:0-ceramide in skeletal muscle was not influenced by time; however, we did observe a tendency for lower C24:0-ceramide in skeletal muscle biopsied from overweight cows. In contrast, we observed a 51% increase in skeletal muscle C16:0-ceramide during the transition from gestation to lactation, albeit no effect of adiposity was detected. In agreement with the observation that ceramide accumulates in skeletal muscle of insulin-resistant humans [61, 62], C16:0-ceramide concentration in skeletal muscle was inversely associated with insulin-stimulated reductions in glucose postpartum (*r* = -0.63, *P* < 0.05). Raichur et al. [48] and Turpin et al. [15] have proposed that skeletal muscle C16:0-ceramide is a proapoptotic mediator of the pathophysiology of insulin resistance in response to high-fat feeding, and in contrast, C24:0-ceramide confers antiapoptotic and proliferative functions. Unfortunately, these studies did not evaluate the potential role of circulating ceramide. Mindful of these uncertainties, we consider that the ability of a specific ceramide moiety to antagonize insulin signaling may be influenced by the compartmental source, which may differ across monogastrics and ruminants. Future studies should directly investigate the ability of circulating and intracellular ceramide to promote defects of insulin signaling in bovine skeletal muscle.

**Fig 6:**
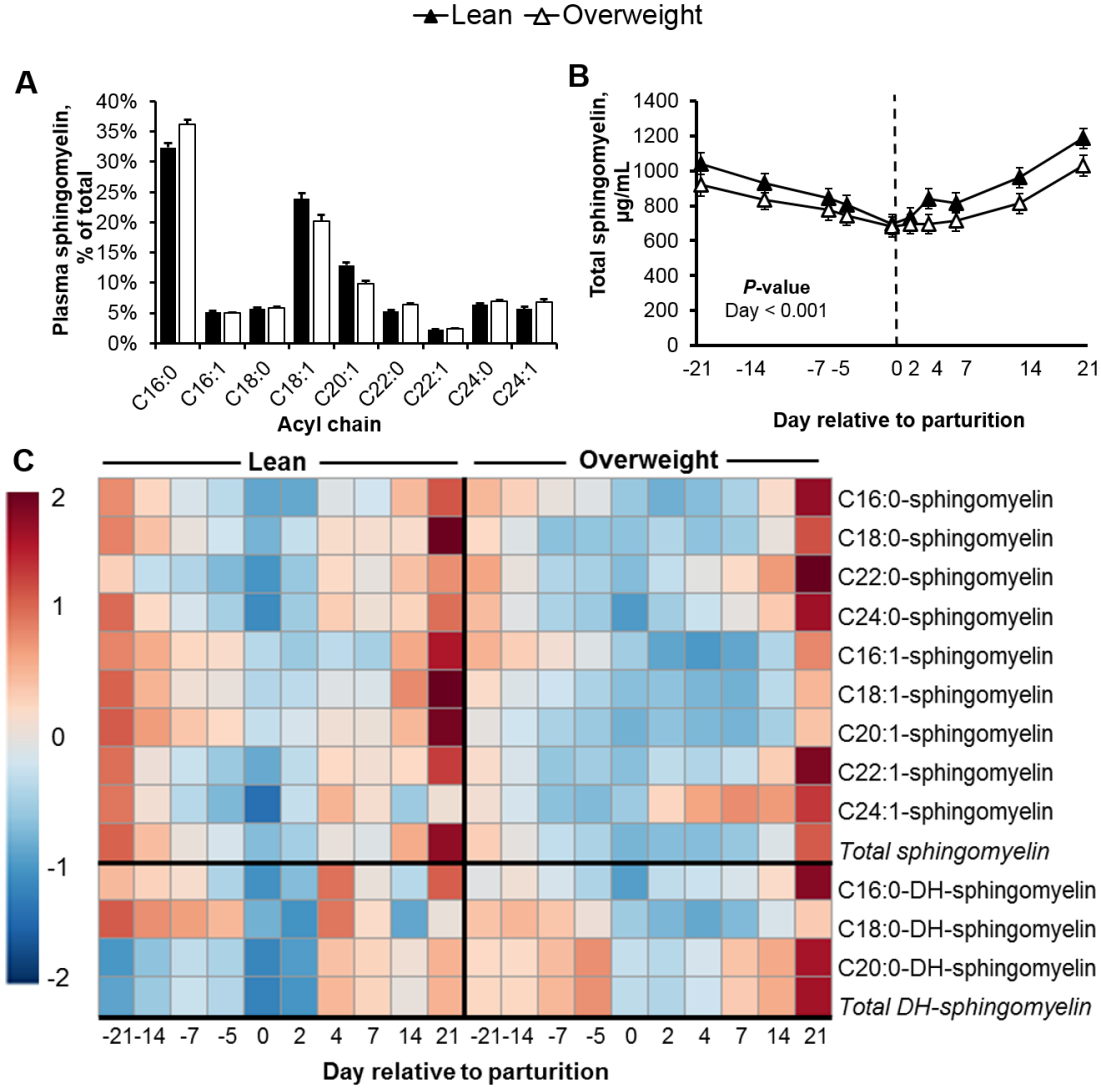
Plasma sphingomyelins reach nadir at parturition. (**A**) Plasma profile of sphingomyelin and (**B**) peripartal changes of plasma total sphingomyelin in lean and overweight cows. Data are represented as least squares means and their standard errors. (**C**) Global changes in plasma sphingomyelins during peripartum. For visualization purposes, the heat map represents fold-change of log2 transformed data. The main effect of day relative to parturition was significant for all sphingolipids shown (*P* < 0.01). The main effect of adiposity (BCS) was significant for C20:1-sphingomyelin (*P* < 0.01). BCS × Day interaction significant for C18:1-sphingomyelin. DH: dihydro.

**Fig 7:**
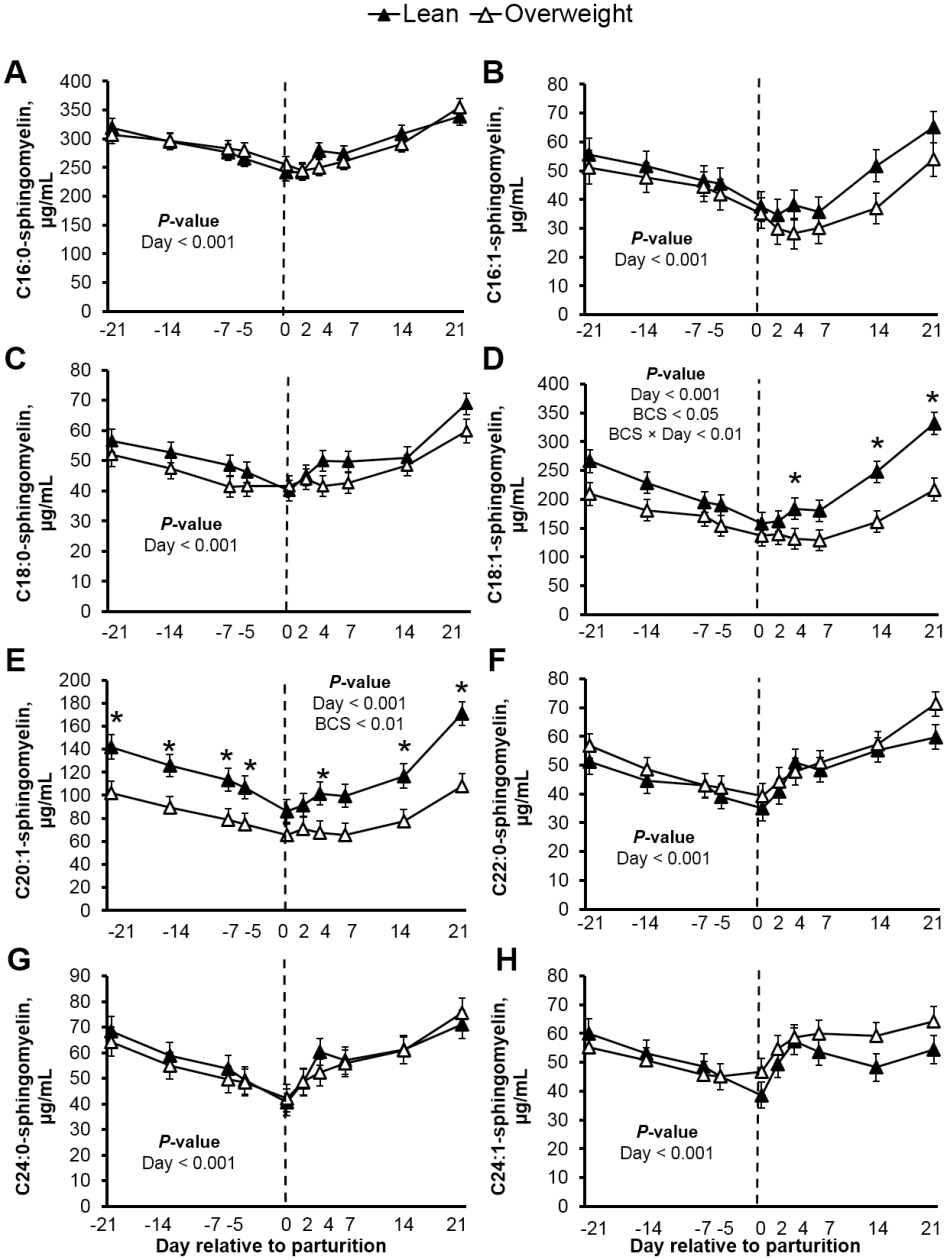
Plasma sphingomyelins decrease toward parturition and increase postpartum. Plasma concentrations (μg/mL) of (**A**) C16:0-, (**B**) C16:1-, (**C**) C18:0-, (**D**) C18:1-, (**E**) C20:0-, (**F**) C22:0-, (**G**) C24:0-, and (**H**) C24:1-sphingomyelin in lean and overweight peripartal dairy cows. Data are represented as least squares means and their standard errors. *, *P* < 0.05; †, *P* < 0.10.

Although de novo ceramide synthesis can contribute to ceramide pools, the activation of sphingomyelinase and subsequent hydrolysis of sphingomyelins to produce ceramide in response to pro-inflammatory signals is well characterized in monogastrics [19, 20], and deserves consideration in cows. Considering that parturition is accompanied by a weakened immune status in dairy cattle [63], we explored changes in circulating sphingomyelins (Figs 6 and 7) and dihydrosphingomyelins (S4 Fig). Similar to adult humans [52], C16:0-, C18:1-, and C20:1-sphingomyelin were the predominant sphingomyelins measured in plasma (Figs 6 and 7). Plasma total sphingomyelin levels reached a nadir at parturition, a response pattern that was observed for all sphingomyelins profiled. Specifically, we observed a 30% decrease in plasma total sphingomyelins at d 0, relative to d -21 prepartum (*P* < 0.001). Similar changes were observed for dihydrosphingomyelins. Although prepartum adiposity did not influence total sphingomyelin concentration, we did detect a significant decrease in C18:1 and C20:1-sphingomyelin levels in overweight cows (Fig 7; *P* < 0.05). Increased adiposity is associated with adipose tissue macrophage infiltration and tumor necrosis factor-α release [64, 65]; therefore, our data supports the possibility that increased adiposity may have predisposed cows to greater inflammation and sphingomyelinase activation. Consistent with sphingomyelin hydrolysis as a source of ceramides, inverse associations between ceramides and sphingomyelins were detected in plasma during prepartum (e.g. *r* = -0.43 for C18:0-linked ceramide and sphingomyelin; *P* < 0.01). Alternatively, the hepatic synthesis and subsequent lipoprotein export of sphingomyelin may have been suppressed because of inadequate phosphocholine supply. In support, we observed a reduction in dry matter intake as parturition approached, which may have limited the intestinal absorption of dietary choline. Of importance because choline is a limiting nutrient in peripartal dairy cow diets [66]. Indeed, we did observe a positive association between dry matter intake and circulating C18:1- and C20:1-sphingomyelin in overweight cows (*r* = 0.72 and 0.67, respectively; *P* < 0.001; S5 Fig), select sphingomyelin moieties that were reduced in overweight cows. It is intriguing to recognize that circulating sphingomyelins represent a concentrated sphingolipid pool (608-fold higher than ceramides), suggesting that minor changes in sphingomyelin levels can dramatically influence ceramide supply. The interplay between sphingomyelin metabolism and the accrual of ceramide during the progression of peripartal insulin resistance in dairy cattle deserves further consideration.

Reduced insulin action and lipolysis represent adaptive mechanisms that facilitate necessary shifts in energy metabolism to support the heightened glucose demands of early lactation. The ability of ceramides to disrupt insulin signaling is clear; however, their role in facilitating the development of insulin resistance in cows transitioning from gestation to lactation requires investigation. Herein, we validated our previous work demonstrating that circulating C24:0-ceramide increases concomitant with the magnitude of lipolysis [25]. Furthermore, we have demonstrated the accrual of hepatic C24:0-ceramide in cows with severe fatty liver deposition, and we have established that liver and plasma ceramide profiles are comparable. Therefore, our data supports the possibility that surplus fatty acids are diverted towards hepatic de novo ceramide synthesis, which in turn facilitates lipoprotein ceramide export. We have also established that C16:0-ceramide accumulates in skeletal muscle during the peripartum, demonstrating that changes in ceramide metabolism are uniquely dependent upon the tissue compartment. Moreover, we observed inverse associations between direct assessments of systemic insulin sensitivity and peripartal concentrations of ceramide and glycosylated ceramide, in support for their putative role in mediating whole-body insulin resistance. Finally, we discovered that temporal modifications in circulating sphingomyelins are influenced by parturition and associated with peripartal feed intake. Our data suggest that ceramides are fundamentally involved in the adaptive metabolic changes associated with the onset of lactation.

## Supporting information

S1 Fig Peripartal changes in circulating glycosylated ceramides. Peripartal plasma concentrations (ng/mL) of monohexosylceramides (GlcCer) in lean and overweight cows. Plasma (**A**) C16:1-GlcCer, (**B**) C18:0-GlcCer, (**C**) C18:1-GlcCer, (**D**) C20:0-GlcCer, (**E**) C22:0-GlcCer, (**F**) C22:1-GlcCer, (**G**) C24:1-GlcCer, and **H**) C26:0-GlcCer. Data are represented as least squares means and their standard errors. *, *P* < 0.05; †, *P* < 0.10.
(TIF)

S2 Fig Peripartal changes in circulating lactosylated ceramides. Peripartal plasma concentrations (ng/mL) of lactosylceramides (LacCer) in lean and overweight cows. Plasma (**A**) C18:0-LacCer, (**B**) C22:0-LacCer, and (**C**) C24:1-LacCer, Data are represented as least squares means and their standard errors. *, *P* < 0.05; †, *P* < 0.10.
(TIF)

S3 Fig De novo synthesized C16:0-dihydroceramide increases in plasma during the transition from gestation to lactation. Data are represented as least squares means and their standard errors. *, *P* < 0.05; †, *P* < 0.10.
(TIF)

S4 Fig Plasma dihydro-(DH)-sphingomyelins reach nadir at parturition and increase postpartum. Plasma concentrations (μg/mL) of (**A**) C16:0-, (**B**) C18:0-, (**C**) C20:0-DH-sphingomyelin, and (**D**) total sphingomyelin in lean and overweight peripartal dairy cows. Data are represented as least squares means and their standard errors. *, *P* < 0.05; †, *P* < 0.10.
(TIF)

S5 Fig Selected plasma sphingomyelins are linearly associated with dry matter intake (DMI) in overweight cows. Regression analysis of DMI with (**A**) C18:1-, and (**B**) C20:1-sphingomyelin in plasma during the transition from gestation to lactation.
(TIF)

## References

[1] Eley RM, Thatcher WW, Bazer FW, Wilcox CJ, Becker RB, Head HH, et al Development of the conceptus in the bovine. J Dairy Sci. 1978; 61:467–473. 10.3168/jds.S0022-0302(78)83622-4 659690

[2] Bell AW, Slepetis R, Ehrhardt RA. Growth and accretion of energy and protein in the gravid uterus during late pregnancy. J. Dairy Sci. 1995; 78:1954–1961. 8550905 10.3168/jds.s0022-0302(95)76821-7

[3] Drackley JK, Dann HM, Douglas GN, Janovick Guretzky NA, Litherland NB, Underwood JP, et al Physiological and pathological adaptations in dairy cows that may increase susceptibility to periparturient diseases and disorders. Ital J Anim Sci. 2005; 4:323–344.

[4] Bell AW. Regulation of organic nutrient metabolism during transition from late pregnancy to early lactation. J Anim Sci. 1995; 73:2804–2819. 8582872 10.2527/1995.7392804x

[5] Bell AW, Bauman DE. Adaptations of glucose metabolism during pregnancy and lactation. J Mammary Gland Biol Neoplasia 1997; 2:265–278. 10882310 10.1023/a:1026336505343

[6] Kerestes M, Faigl V, Kulcsár M, Balogh O, Földi J, Fébel H, et al Periparturient insulin secretion and whole‐body insulin responsiveness in dairy cows showing various forms of ketone pattern with or without puerperal metritis. Domest Anim Endocrinol. 2009; 37:250–261. 19716674 10.1016/j.domaniend.2009.07.003

[7] Zachut M, Honig H, Striem S, Zick Y, Boura-Halfon S, Moallem U. Periparturient dairy cows do not exhibit hepatic insulin resistance, yet adipose‐specific insulin resistance occurs in cows prone to high weight loss. J Dairy Sci. 2013; 96:5656–5669. 10.3168/jds.2012-6142 23871373

[8] Mann S, Yepes FA, Duplessis M, et al Dry period plane of energy: Effects on glucose tolerance in transition dairy cows. J Dairy Sci. 2016; 99:701–717. 10.3168/jds.2015-9908 26627861

[9] Summers SA. Ceramides in insulin resistance and lipotoxicity. Prog Lipid Res. 2006; 45:42–72. 10.1016/j.plipres.2005.11.002 16445986

[10] Hannun YA, Obeid LM. Principles of bioactive lipid signalling: lessons from sphingolipids. Nat Rev Mol Cell Biol. 2008; 9:139–150. 10.1038/nrm2329 18216770

[11] Summers SA, Garza LA, Zhou H, and Birnbaum MJ. Regulation of insulin-stimulated glucose transporter GLUT4 translocation and Akt kinase activity by ceramide. Mol Cell Biol. 1998; 18:5457–5464. 9710629 10.1128/mcb.18.9.5457 PMC109130

[12] Stratford S, Hoehn KL, Liu F, Summers SA. Regulation of insulin action by ceramide: dual mechanisms linking ceramide accumulation to the inhibition of Akt/protein kinase B. J Biol Chem. 2004; 279:36608–36615. 10.1074/jbc.M406499200 15220355

[13] Canals D, Perry DM, Jenkins RW, Hannun YA. Drug targeting of sphingolipid metabolism: sphingomyelinases and ceramidases. Br J Pharmacol. 2011; 163:694–712. 10.1111/j.1476-5381.2011.01279.x 21615386 PMC3111673

[14] Chavez JA, Siddique MM, Wang ST, Ching J, Shayman JA, Summers SA. Ceramides and glucosylceramides are independent antagonists of insulin signaling. J Biol Chem. 2014; 289:723–734. 10.1074/jbc.M113.522847 24214972 PMC3887200

[15] Turpin SM, Nicholls HT, Willmes DM, Mourier A, Brodesser S, Wunderlich CM, et al Obesity-induced CerS6-dependent C16:0 ceramide production promotes weight gain and glucose intolerance. Cell Metab. 2014; 20:678–686. 10.1016/j.cmet.2014.08.002 25295788

[16] Watt MJ, Barnett AC, Bruce CR, Schenk S, Horowitz JF, Hoy AJ. Regulation of plasma ceramide levels with fatty acid oversupply: Evidence that the liver detects and secretes de novo synthesised ceramide. Diabetologia. 2012; 55:2741–2746. 10.1007/s00125-012-2649-3 22854889 PMC3576922

[17] Merrill AH Jr. De novo sphingolipid biosynthesis: a necessary, but dangerous, pathway. J Biol Chem. 2002; 277:25843–25846. 10.1074/jbc.R200009200 12011104

[18] Boon J, Hoy AJ, Stark R, Brown RD, Meex RC, Henstridge DC, et al Ceramides contained in LDL are elevated in type 2 diabetes and promote inflammation and skeletal muscle insulin resistance. Diabetes. 2013; 62:401–410. 10.2337/db12-0686 23139352 PMC3554351

[19] Kolesnick R, Golde DW. The sphingomyelin pathway in tumor necrosis factor and interleukin-1 signaling. Cell. 1994; 77:325–328. 8181053 10.1016/0092-8674(94)90147-3

[20] Peraldi P, Hotamisligil GS, Buurman WA, White MF, Spiegelman B.M. Tumor necrosis factor (TNF)-alpha inhibits insulin signaling through stimulation of the p55 TNF receptor and activation of sphingomyelinase. J Biol Chem. 1996; 271:13018–13022. 8662983 10.1074/jbc.271.22.13018

[21] Summers SA, Goodpaster BH. CrossTalk proposal: Intramyocellular ceramide accumulation does modulate insulin resistance. J Physiol. 2016; 594:3167–3170. 10.1113/JP271676 26996141 PMC4800028

[22] Petersen MC, Jurczak MJ. CrossTalk opposing view: Intramyocellular ceramide accumulation does not modulate insulin resistance. J Physiol. 2016; 594:3171–3174. 10.1113/JP271677 26997581 PMC4908018

[23] Holland WL, Brozinick JT, Wang LP, Hawkins ED, Sargent KM, Liu Y, et al Inhibition of ceramide synthesis ameliorates glucocorticoid-, saturated-fat-, and obesity-induced insulin resistance. Cell Metab. 2007; 5:167–179. 10.1016/j.cmet.2007.01.002 17339025

[24] Pagadala M, Kasumov T, McCullough AJ, Zein NN, Kirwan JP. Role of ceramides in nonalcoholic fatty liver disease. Trends Endocrinol Metab. 2012; 23:365–371. 10.1016/j.tem.2012.04.005 22609053 PMC3408814

[25] Rico JE, Bandaru VVR, Dorskind JM, Haughey NJ, McFadden JW. Plasma ceramides are elevated in overweight Holstein dairy cows experiencing greater lipolysis and insulin resistance during the transition from late pregnancy to early lactation. J Dairy Sci. 2015; 98:7757–70. 10.3168/jds.2015-9519 26342987 PMC6075710

[26] Rico JE, Mathews AT, Lovett J, Haughey NJ, McFadden JW. Palmitic acid feeding increases ceramide supply in association with increased milk yield, circulating nonesterified fatty acids, and adipose tissue responsiveness to a glucose challenge. J Dairy Sci. 2016; 99:8817–8830. 10.3168/jds.2016-11296 27638262

[27] Haus JM, Kashyap SR, Kasumov T, Zhang R, Kelly KR, DeFronzo RA, et al Plasma ceramides are elevated in obese subjects with type 2 diabetes and correlate with the severity of insulin resistance. Diabetes. 2009; 58:337–343. 10.2337/db08-1228 19008343 PMC2628606

[28] Wildman EE, Jones GM, Wagner PE, Bowman RL. A dairy cow body condition scoring system and its relationship to selected production characteristics. J Dairy Sci. 1982; 65:495–501.

[29] National Research Council—NRC. Nutrient Requirements of Dairy Cattle 7th rev. ed Natl Acad Sci. Washington. DC 2001.

[30] Hughes JP. A simplified instrument for obtaining liver biopsies in cattle. Am J Vet Res. 1962; 23:1111–1113. 14449814

[31] Pires J. A., Souza A. H., and Grummer R. R.. Induction of hyperlipidemia by intravenous infusion of tallow emulsion causes insulin resistance in Holstein cows. J. Dairy Sci. 2007; 90:2735–2744. 10.3168/jds.2006-759 17517713

[32] Van Soest PJ, Robertson JB, Lewis BA. Methods for dietary fiber, neutral detergent fiber, and nonstarch polysaccharides in relation to animal nutrition. J. Dairy Sci. 1991; 74:3583–3597. 10.3168/jds.S0022-0302(91)78551-2 1660498

[33] AOAC. AOAC International. Official Methods of Analysis. 17th ed AOAC International 2000 Gaithersburg, MD, USA.

[34] Hall MB. Determination of starch, including maltooligosaccharides, in animal feeds: Comparison of methods and a method recommended for aoac collaborative study. J AOAC Int. 2009; 92:42–49. 19382561

[35] AOAC. AOAC Official Methods of Analysis. 19th ed AOAC International 2012 Gaithersburg, MD, USA.

[36] Starke A, Haudum A, Busche R, Beyerbach M, Dänicke S, Rehage J. Technical note: Analysis of total lipid and triacylglycerol content in small liver biopsy samples in cattle. J Anim Sci. 2010; 88:2741–2750. 10.2527/jas.2009-2599 20348378

[37] Haughey NJ, Cutler RG, Tamara A, McArthur JC, Vargas DL, Pardo CA, et al Perturbation of sphingolipid metabolism and ceramide production in HIV-dementia. Ann. Neurol. 2004; 55:257–267. 10.1002/ana.10828 14755730

[38] Xia J, Sinelnikov I, Han B, Wishart DS. MetaboAnalyst 3.0—making metabolomics more meaningful. Nucleic Acids Res. 2015; 43:W251–257. 10.1093/nar/gkv380 25897128 PMC4489235

[39] Morrow DA. Fat cow syndrome. J Dairy Sci. 1976; 59:1625–1629. 10.3168/jds.S0022-0302(76)84415-3 965540

[40] Roche JR, Friggens NC, Kay JK, Fisher MW, Stafford KJ, Berry DP. Invited review: Body condition score and its association with dairy cow productivity, health, and welfare. J. Dairy Sci. 2009; 92:5769–5801. 10.3168/jds.2009-2431 19923585

[41] Bauman DE, Currie WB. Partitioning of nutrients during pregnancy and lactation: a review of mechanisms involving homeostasis and homeorhesis. J Dairy Sci. 1980; 63:1514–1529. 7000867 10.3168/jds.s0022-0302(80)83111-0

[42] Chapinal N, Leblanc SJ, Carson ME, Leslie KE, Godden S, Capel M, et al Herd-level association of serum metabolites in the transition period with disease, milk production, and early lactation reproductive performance. J Dairy Sci. 2012; 95:5676–5682. 10.3168/jds.2011-5132 22863094

[43] Drackley JK. ADSA Foundation Scholar Award. Biology of dairy cows during the transition period: the final frontier? J Dairy Sci. 1999; 82:2259–2273. 10575597 10.3168/jds.s0022-0302(99)75474-3

[44] Ohtsuka H, Koiwa M, Hatsugaya A, Kudo K, Hoshi F, Itoh N, et al Relationship between serum TNF activity and insulin resistance in dairy cows affected with naturally occurring fatty liver. J Vet Med Sci. 2001; 63:1021–1025. 11642272 10.1292/jvms.63.1021

[45] Oikawa S, Oetzel GR. Decreased insulin response in dairy cows following a four-day fast to induce hepatic lipidosis. J Dairy Sci. 2006; 89:2999–3005. 10.3168/jds.S0022-0302(06)72572-3 16840615

[46] Bobe G, Young JW, Beitz DC. Invited review: pathology, etiology, prevention, and treatment of fatty liver in dairy cows. J Dairy Sci. 2004; 87:3105–3124. 10.3168/jds.S0022-0302(04)73446-3 15377589

[47] Kasumov T, Li L, Li M, Gulshan K, Kirwan JP, Liu X, et al Ceramide as a mediator of non-alcoholic Fatty liver disease and associated atherosclerosis. PLoS One. 2015; 10:e0126910 10.1371/journal.pone.0126910 25993337 PMC4439060

[48] Raichur S, Wang ST, Chan PW, Li Y, Ching J, Chaurasia B, et al CerS2 haploinsufficiency inhibits β-oxidation and confers susceptibility to diet-induced steatohepatitis and insulin resistance. Cell Metab. 2014; 20:684–695. 10.1016/j.cmet.2014.10.007 29665397

[49] Levy M, Futerman AH. 2010 Mammalian ceramide synthases. IUBMB Life 62:347–356. 10.1002/iub.319 20222015 PMC2858252

[50] Mathews AT, Rico JE, Sprenkle NT, Lock AL, McFadden JW. Increasing palmitic acid intake enhances milk production and prevents glucose-stimulated fatty acid disappearance without modifying systemic glucose tolerance in mid-lactation dairy cows. J Dairy Sci. 2016; 99:8802–8816. 10.3168/jds.2016-11295 27638259

[51] Park M, Kaddai V, Ching J, Fridianto KT, Sieli RJ, Sugii S, et al A Role for Ceramides, but Not Sphingomyelins, as Antagonists of Insulin Signaling and Mitochondrial Metabolism in C2C12 Myotubes. J Biol Chem. 2016; 291:23978–23988. 10.1074/jbc.M116.737684 27703011 PMC5104923

[52] Hanamatsu H, Ohnishi S, Sakai S, Yuyama K, Mitsutake S, Takeda H, et al Altered levels of serum sphingomyelin and ceramide containing distinct acyl chains in young obese adults. Nutr Diabetes. 2014; 4:e141 10.1038/nutd.2014.38 25329603 PMC4217001

[53] Kabayama K, Sato T, Saito K, Loberto N, Prinetti A, Sonnino S, et al Dissociation of the insulin receptor and caveolin-1 complex by ganglioside GM3 in the state of insulin resistance. Proc Natl Acad Sci U S A. 2007; 104:13678–13683. 10.1073/pnas.0703650104 17699617 PMC1949342

[54] Kabayama K, Sato T, Kitamura F, Uemura S, Kang BW, Igarashi Y, et al TNFalpha-induced insulin resistance in adipocytes as a membrane microdomain disorder involvement of ganglioside GM3. Glycobiology. 2004; 15:21–29. 10.1093/glycob/cwh135 15306563

[55] Aerts JM, Ottenhoff R, Powlson AS, Grefhorst A, van Eijk M, Dubbelhuis PF, et al Pharmacological inhibition of glucosylceramide synthase enhances insulin sensitivity. 2007 Diabetes; 56:1341–1349. 10.2337/db06-1619 17287460 PMC4298701

[56] Xia JY, Holland WL, Kusminski CM, Sun K, Sharma AX, Pearson MJ, et al Targeted Induction of Ceramide Degradation Leads to Improved Systemic Metabolism and Reduced Hepatic Steatosis. Cell Metab. 2015; 22:266–278. 10.1016/j.cmet.2015.06.007 26190650 PMC4527941

[57] Hla T, Kolesnick R. C16:0-ceramide signals insulin resistance. Cell Metab. 2014; 20:703–705. 10.1016/j.cmet.2014.10.017 25440051 PMC4393079

[58] Ghafourifar P, Klein SD, Schucht O, Schenk U, Pruschy M, Rocha S, et al Ceramide induces cytochrome c release from isolated mitochondria. Importance of mitochondrial redox state. J Biol Chem. 1999; 274:6080–6084. 10037689 10.1074/jbc.274.10.6080

[59] Blouin CM, Prado C, Takane KK, Lasnier F, Garcia-Ocana A, Ferré P, et al Plasma membrane subdom.ain compartmentalization contributes to distinct mechanisms of ceramide action on insulin signaling. Diabetes. 2010; 59:600–610. 10.2337/db09-0897 19959757 PMC2828662

[60] Powell DJ, Hajduch E, Kular G, Hundal HS: Ceramide disables 3-phosphoinositide binding to the pleckstrin homology domain of protein kinase B (PKB)/Akt by a PKCzeta-dependent mechanism. Mol Cell Biol. 2003; 23:7794–7808. 10.1128/MCB.23.21.7794-7808.2003 14560023 PMC207567

[61] Adams JM, Pratipanawatr T, Berria R, Wang E, DeFronzo RA, Sullards MC, et al Ceramide content is increased in skeletal muscle from obese insulin-resistant humans. Diabetes. 2004; 53:25–31. 14693694 10.2337/diabetes.53.1.25

[62] Straczkowski M, Kowalska I, Baranowski M, Nikolajuk A, Otziomek E, Zabielski P, et al Increased skeletal muscle ceramide level in men at risk of developing type 2 diabetes. Diabetologia. 2007; 50:2366–2373. 10.1007/s00125-007-0781-2 17724577

[63] Sordillo L.M., and Raphael W.. Significance of metabolic stress, lipid mobilization, and inflammation on transition cow disorders. Vet Clin North Am Food Anim Pract. 2013; 29:267–78. 10.1016/j.cvfa.2013.03.002 23809891

[64] Contreras GA, Kabara E, Brester J, Neuder L, Kiupel M. Macrophage infiltration in the omental and subcutaneous adipose tissues of dairy cows with displaced abomasum. J Dairy Sci; 2015; 98:6176–6187. 10.3168/jds.2015-9370 26117355

[65] O'Boyle N, Corl CM, Gandy JC, Sordillo LM. Relationship of body condition score and oxidant stress to tumor necrosis factor expression in dairy cattle. Vet Immunol Immunopathol. 2006; 113:297–304. 10.1016/j.vetimm.2006.05.013 16842861

[66] Cooke RF, Silva Del Rio N, Caraviello DZ, Bertics SJ, Ramos MH, Grummer RR. Supplemental choline for prevention and alleviation of fatty liver in dairy cattle. J Dairy Sci. 2007; 90:2413–2418. 10.3168/jds.2006-028 17430945

