# Supplementary figures and images for: Temporal changes in sphingolipids and systemic insulin sensitivity during the transition from gestation to lactation
Source: PLoS One. 2017 May 9;12(5):e0176787. doi: 10.1371/journal.pone.0176787 (PMC5423608; doi:10.1371/journal.pone.0176787)

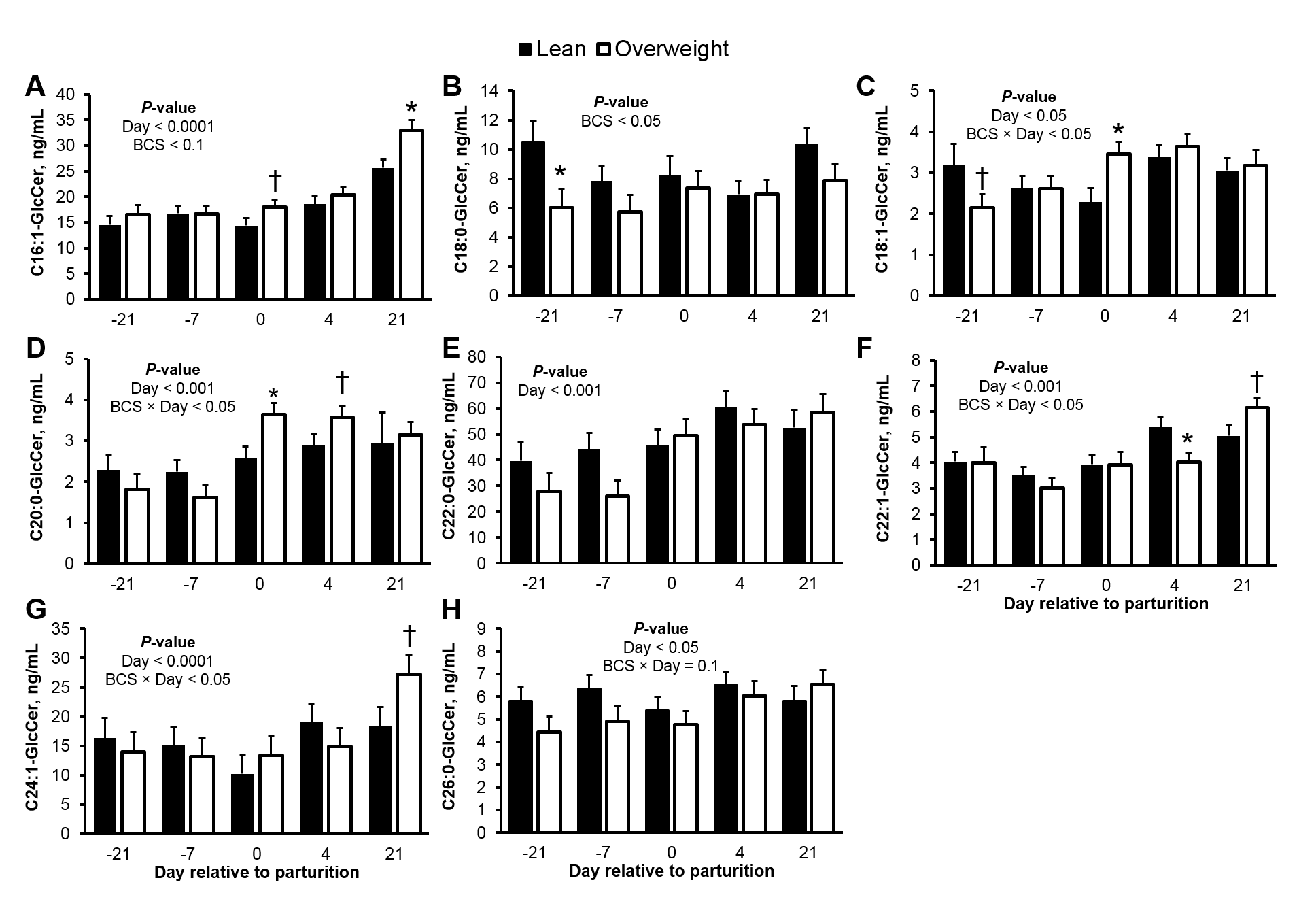

Supplement: S1 Fig — Peripartal plasma concentrations (ng/mL) of monohexosylceramides (GlcCer) in lean and overweight cows. Plasma (A) C16:1-GlcCer, (B) C18:0-GlcCer, (C) C18:1-GlcCer, (D) C20:0-GlcCer, (E) C22:0-GlcCer, (F) C22:1-GlcCer, (G) C24:1-GlcCer, and H) C26:0-GlcCer. Data are represented as least squares means and their standard errors. *, P < 0.05; †, P < 0.10. (TIF) [file pone.0176787.s001.tif]

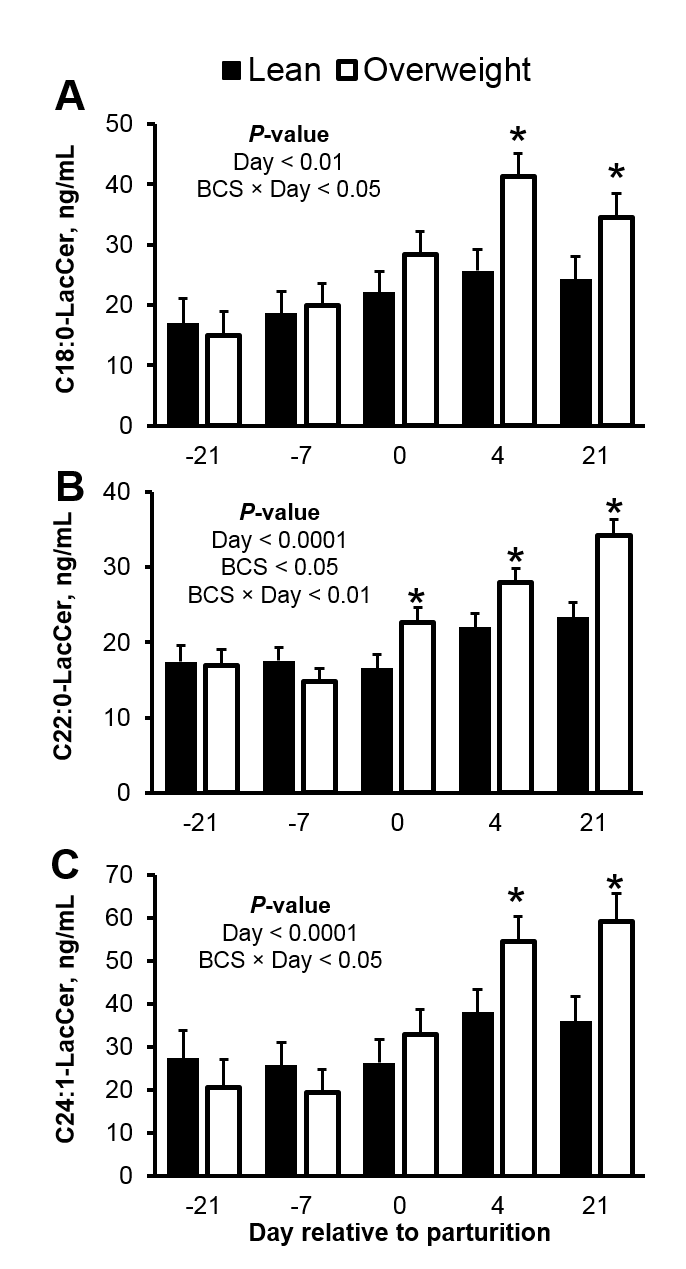

Supplement: S2 Fig — Peripartal plasma concentrations (ng/mL) of lactosylceramides (LacCer) in lean and overweight cows. Plasma (A) C18:0-LacCer, (B) C22:0-LacCer, and (C) C24:1-LacCer, Data are represented as least squares means and their standard errors. *, P < 0.05; †, P < 0.10. (TIF) [file pone.0176787.s002.tif]

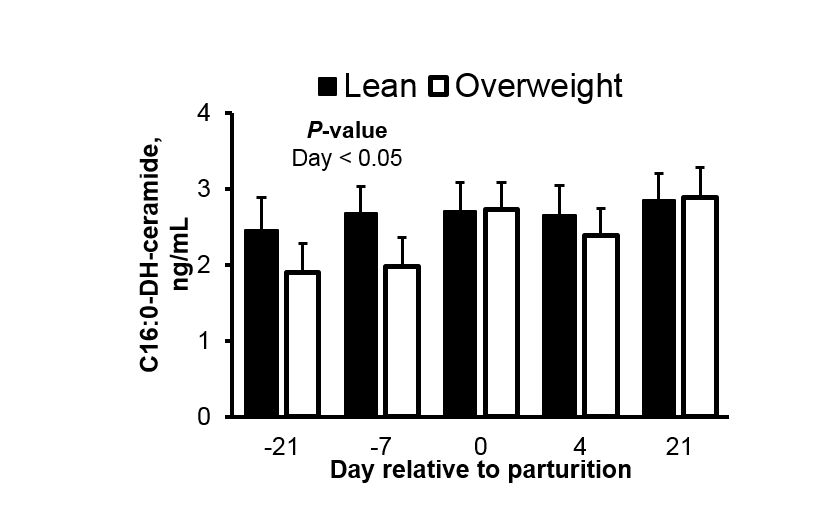

Supplement: S3 Fig — Data are represented as least squares means and their standard errors. *, P < 0.05; †, P < 0.10. (TIF) [file pone.0176787.s003.tif]

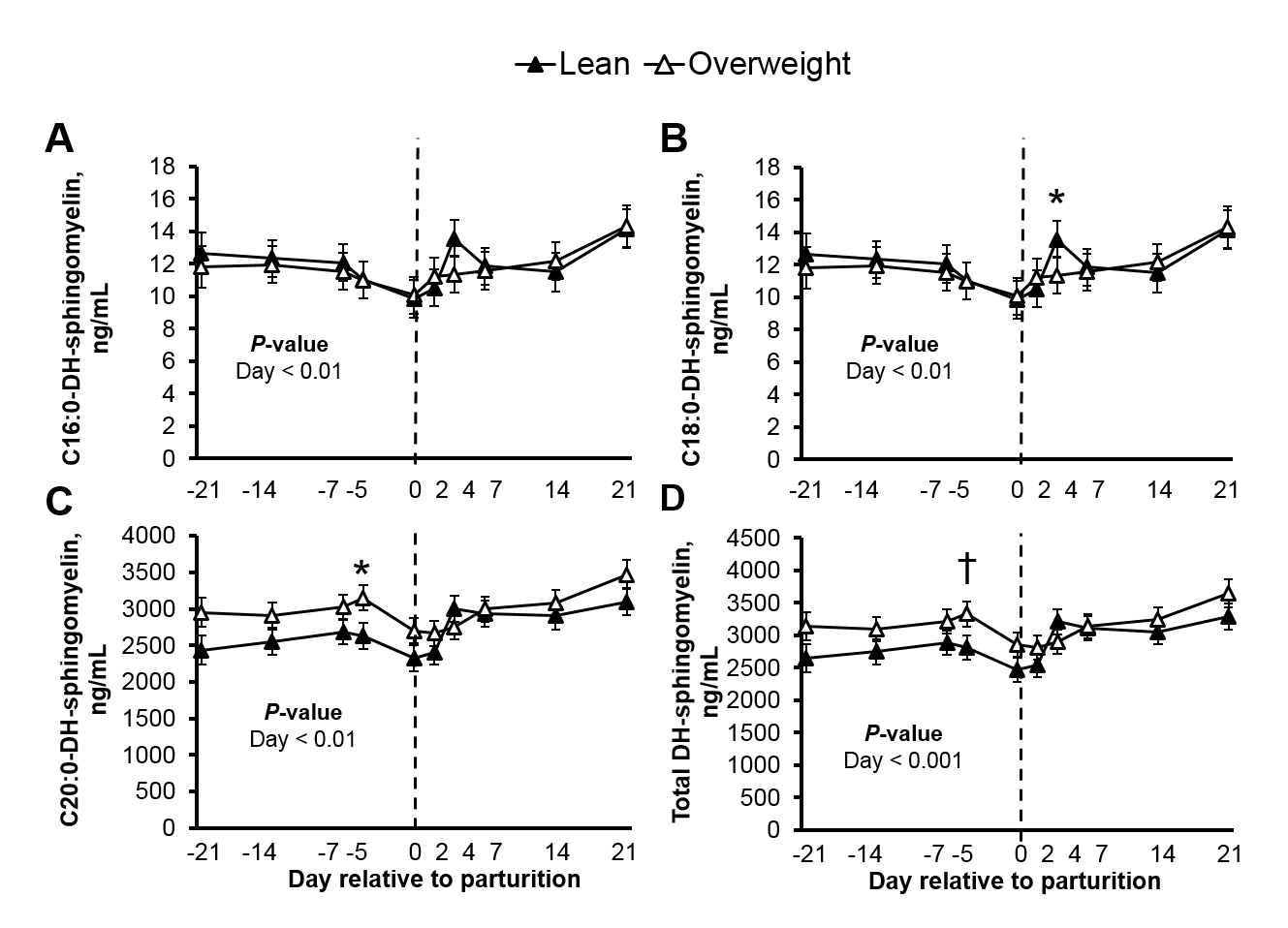

Supplement: S4 Fig — Plasma concentrations (μg/mL) of (A) C16:0-, (B) C18:0-, (C) C20:0-DH-sphingomyelin, and (D) total sphingomyelin in lean and overweight peripartal dairy cows. Data are represented as least squares means and their standard errors. *, P < 0.05; †, P < 0.10. (TIF) [file pone.0176787.s004.tif]

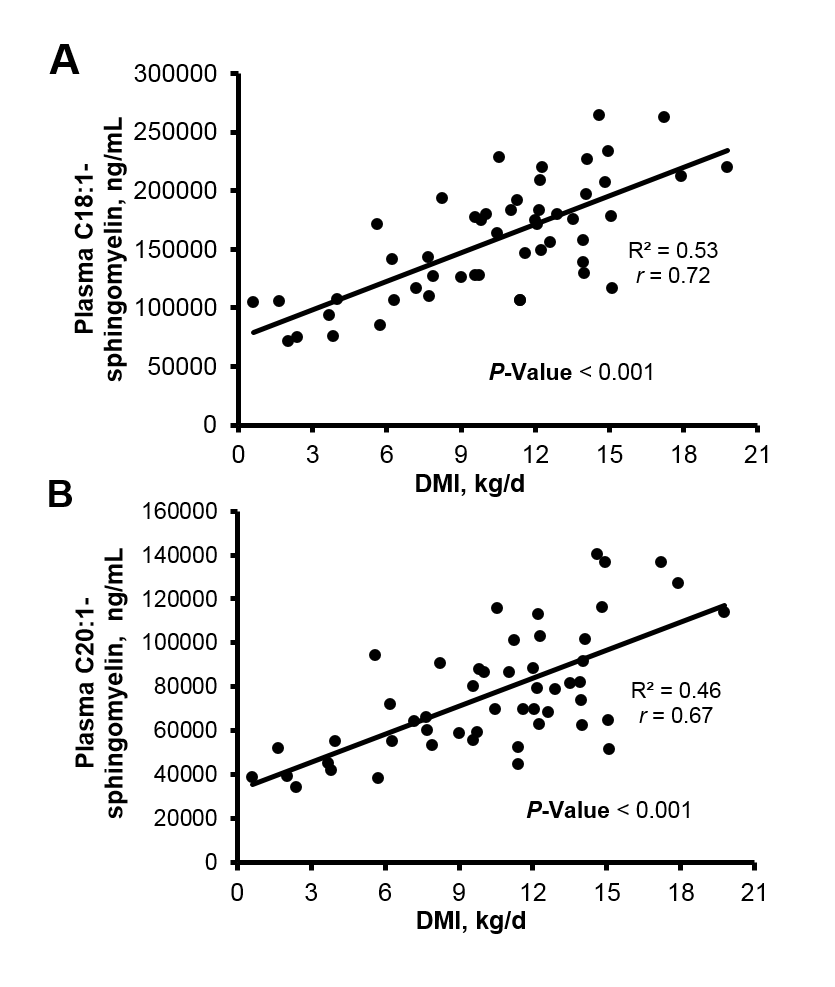

Supplement: S5 Fig — Regression analysis of DMI with (A) C18:1-, and (B) C20:1-sphingomyelin in plasma during the transition from gestation to lactation. (TIF) [file pone.0176787.s005.tif]
